# Extract of Seaweed *Codium fragile* Inhibits Integrin αIIbβ3-Induced Outside-in Signaling and Arterial Thrombosis

**DOI:** 10.3389/fphar.2021.685948

**Published:** 2021-07-02

**Authors:** Tae In Kim, Yeon-Ji Kim, Kyungho Kim

**Affiliations:** Korean Medicine-Application Center, Korea Institute of Oriental Medicine, Daegu, South Korea

**Keywords:** platelet, thrombosis, outside-in signaling, integrin αIIbβ3, codium fragile

## Abstract

Seaweeds are thought to be promising candidates for functional foods and to help prevent thrombotic and related cardiovascular diseases. *Codium fragile* (Suringer) Hariot has been traditionally used as a culinary ingredient, and it possesses a range of biological activities, including the inhibition of platelet function. However, the mechanism of this inhibition is unclear. The aim of this study was to examine the inhibitory effect of *C. fragile* in platelet function. The antiplatelet activity of *C. fragile* on agonist-activated platelet aggregation, granule secretion, calcium mobilization, platelet spreading, and clot retraction was assessed. The phosphorylation of c-Src, Syk, PLCγ2, and several proteins involving in the αIIbβ3 integrin outside-in signaling pathway were also studied in thrombin and CRP-stimulated platelets. The antithrombotic effect was investigated in mice using ferric chloride-induced arterial thrombus formation *in vivo*. Transection tail bleeding time was used to evaluate whether *C. fragile* inhibited primary hemostasis. The main components and contents of *C. fragile* ethanol extract were confirmed by GC-MS analysis. *C. fragile* significantly impaired agonist-induced platelet aggregation granule secretion, calcium mobilization, platelet spreading, and clot retraction. Biochemical analysis revealed that *C. fragile* inhibited the agonist-induced activation of c-Src, Syk, and PLCγ2, as well as the phosphorylation of PI3K, AKT, and mitogen-activated protein kinases (MAPKs). The inhibitory effect of *C. fragile* resulted from an inhibition of platelet αIIbβ3 integrin outside-in signal transduction during cell activation. Oral administration of *C. fragile* efficiently blocked FeCl_3_-induced arterial thrombus formation *in vivo* without prolonging bleeding time. GC-MS analysis revealed that phytol was the main constituent and the total content of isomers was 160.8 mg/kg. Our results demonstrated that *C. fragile* suppresses not only the inside-out signaling of αIIbβ3 integrin but also outside-in signal transmission. Therefore, *C. fragile* could be an effective antiplatelet therapeutic candidate.

## Introduction

Platelets play crucial roles in thrombosis and hemostasis. Platelet integrin αIIbβ3 is a key mediator of platelet aggregation and is abundantly expressed on the platelet surface (Shen et al., 2013). In resting platelets, integrin αIIbβ3 is maintained in a low-affinity binding state, in which the extracellular domain of αIIbβ3 integrin is in a closed conformation. However, upon the activation of a platelet, αIIbβ3 undergoes a conformational change from a low-affinity state to a high-affinity ligand-binding state, in which it can bind ligands such as fibrinogen (FG) and von Willebrand factor (Takagi et al., 2002; Bennett, 2005; Li et al., 2010). Ligand binding to activated integrin αIIbβ3 induces a cascade of outside-in signaling events, thereby facilitating platelet spreading, aggregation, clot retraction, and thrombosis (Takagi et al., 2002; Gong et al., 2010).

Outside-in signaling *via* platelet integrin αIIbβ3 involves a wide range of enzymes, signaling adaptors, and cytoskeletal components (Estevez et al., 2015). The integrin αIIbβ3-induced platelet outside-in signaling pathway is initiated by members of the sarcoma tyrosine-protein kinase (c-Src) family of kinases (SFKs)-mediated phosphorylation events. Cellular and c-Src is associated with the cytoplasmic tail of β3 integrin and activated by the signaling cascades involved in the recruitment and activation of focal adhesion kinase (FAK), phosphoinositide 3-kinase (PI3K), and protein kinase B (AKT) (Arias-Salgado et al., 2003; Li et al., 2010; Durrant et al., 2017). Ligand binding to integrin αIIbβ3 also triggers the tyrosine phosphorylation of signaling cascades involved in the recruitment and activation of spleen tyrosine kinase (Syk), phospholipase Cγ2 (PLCγ2), and SH2 domain-containing leukocyte protein of 76 kDa (SLP-76), the proto-oncogene vav (Vav1), PI3K, and more, thereby initiating downstream platelet responses, such as granule secretion, platelet spreading, and clot retraction (Law et al., 1999; Phillips et al., 2001; Wonerow et al., 2003; Suzuki-Inoue et al., 2007b).

The ligand-binding function of integrin αIIbβ3 has been considered as a potential target for the development of antithrombotic agents (Estevez et al., 2015; Xu et al., 2016). However, current antithrombotic agents have significant side effects against thrombocytopenia and increase the risk of bleeding (Alexander and Peterson, 2010). These side effects limit the applicability and dosage of the antithrombotic agents, thereby restricting their effectiveness (Wang et al., 2014b; Xu et al., 2016). Recently, studies have suggested that selectively targeting the integrin αIIbβ3-induced platelet outside-in signaling pathway allows for strong inhibition of thrombosis without hemostasis (Estevez et al., 2015). The main advantage of targeting αIIbβ3 integrin-mediated platelet outside-in signaling is that intervention in this pathway does not affect primary platelet adhesion and aggregation, which are important for hemostasis, but limits the size of thrombus formation, which prevents vascular occlusion (Estevez et al., 2015). Thus, selective inhibitors of integrin outside-in signaling could be potential new antithrombotic drugs.

Cardiovascular disease (CVD) is the leading cause of global mortality and morbidity. There are several risk factors associated with CVD, including high cholesterol, diet, hypertension, atherosclerosis, and thrombosis (Falk, 2006; Saleh Al-Shehabi et al., 2016). The healing properties of natural products have long been identified as one of the most important strategies for treating and managing CVD (Al Disi et al., 2015; Shaito et al., 2020). Recently, the interest in natural products including medicinal herbs has been increased based on the effectiveness against CVD (Shaito et al., 2020). Due to the wide range of biological activities, natural products offer a promise to develop novel pharmacological agents that may prove promising in controlling CVD. Edible marine algae are considered to be good sources of nutrients, and the algae have diverse biological activities, including anti-inflammatory, anti-oxidative, anti-cancer, and anti-nociceptive effects (Wang et al., 2014a). *Codium fragile* (Suringer) Hariot is a heavily utilized edible green alga belonging to the family Codiaceae. The algae are widely distributed along the coasts of East Asia, Oceania, and Northern Europe. In Korea, *C. fragile* has been used as a culinary ingredient and traditional medicine to treat enterobiasis, dropsy, and dysuria (Lehnhardt Pires et al., 2013). Several studies have indicated a protective effect of *C. fragile* against pro-inflammatory stimuli, oxidative damage, and tumor progression in experimental models (Kang et al., 2012; Lee et al., 2013; Dilshara et al., 2016). Sterol-based compounds with varying bioactivities were identified as the main components of *C. fragile* extract (Rubinstein and John Goad, 1974; Lee et al., 2013). However, the protective effect and molecular mechanisms underlying the potential inhibitory effects of *C. fragile* on platelet thrombus formation have not been fully elucidated. Therefore, in the present study, we aimed to clarify whether *C. fragile* is involved in the attenuation of platelet function and integrin αIIbβ3 signaling and to identify which compounds produce antiplatelet activity in *C. fragile*.

In the present study, we found that extract of *C. fragile* inhibited thrombus formation *in vivo* and *in vitro*. *C. fragile* specifically inhibited platelet activation and aggregation induced by thrombin, collagen, collagen-related peptide (CRP), adenosine diphosphate (ADP), and U46619 (thromboxane A2 analogue). Using biochemical approaches, we found that the phosphorylation levels of the c-Src/Syk/PLCγ2/PI3K/AKT/MAPK axis were inhibited by treatment with *C. fragile*. GC-MS analysis was conducted to identify and quantify the main constituents of *C. fragile* extract. *C. fragile* inhibited platelet spreading on immobilized FG and clot retraction. These findings suggest that *C. fragile* regulates integrin αIIbβ3-mediated outside-in signaling by the inhibition of platelet activation. Studies using a mouse model of FeCl_3_-induced arterial thrombosis indicated that *C. fragile* plays a crucial role in arterial thrombosis. The tail bleeding time was not significantly higher in *C. fragile*-treated mice than in control mice. These results demonstrate that *C. fragile* can potentially exert antiplatelet and antithrombotic effects without affecting hemostasis.

## Materials and Methods

Reagents. Human thrombin, PGE1, rhodamine-phalloidin, dimethyl sulfoxide (DMSO), ADP, fibrinogen, human fibrinogen, ferric chloride (FeCl_3_), Acetylsalicylic acid (ASA), and all the reagents were purchased from Sigma (St. Louis, MO, United States). Equine tendon collagen (type I) and ATP luciferin/luciferase reagent were obtained from Chrono-log (Havertown, PA). D-Phe-Pro-Arg-chloromethyl ketone (PPACK) was purchased from EMD Millipore (Billerica, MA, United States). CRP was obtained from Dr Richard Farndale (Department of Biochemistry, University of Cambridge, United Kingdom). Phycoerythrin (PE)-conjugated isotype control IgGs, rat monoclonal antibodies against mouse P-selectin, activated αIIbβ3 (JON/A) were from Emfret Analytics (Eibelstadt, Germany). Antibodies against phospho-c-Src at Tyr416, phospho-Syk at Tyr525/526, phospho-PLCγ2 at Tyr759, phospho-PLCγ2 at Tyr1217, phospho-PI3K p85α/β at Tyr458/p55α/γ at Tyr199, phospho-Akt at Ser473, phospho-p38 at Thr180/Tyr182, phospho-ERK at Thr202/Tyr204, phosphor-FAK at Tyr397, Total c-Src, Total Syk, Total PLCγ2, Total PI3K p85, Total Akt, Total p38, Total ERK, Total FAK, and actin were obtained from Cell Signaling (Danvers, MA, United States). Monoclonal antibodies against phospho-integrin β3 at Tyr 759 and Total integrin β3 were obtained from Santa Cruz (Santa Cruz, CA, United States). Calcium dye (FLIPR Calcium Assay kit) was from Molecular Devices (Sunnyvale, CA, United States). Phytol (a mixture of isomers) was purchased from Sigma (St. Louis, MO, United States). HPLC-grade methanol was obtained from JT Baker (Philipsburg, NJ, United States).

*C. fragile* preparation. The *C. fragile* was collected from the Wando Island coast of Korea, and its identity was confirmed by Dr Wei Li. A voucher specimen (KIOM-30) was deposited at the Herbarium of Korean Medicine-Application Center, Korea Institute of Oriental Medicine, Republic of Korea. The collected *C. fragile* was soaked in fresh water for a day to remove salt and dry at 65°C. The dried *C. fragile* (50.0 g) was pulverized and extracted with 1 L of 70% EtOH or 1 L of water at room temperature for 2 weeks. The filtrate was evaporated and freeze-dried (22.86 g, yield 45.7%) and the dry extract powder was stored at 4°C until used (Supplementary Figure S1A).

Instrumentation. The GC-MS analysis was conducted using the Shimadzu GC-MS-QP2010 Ultra system (GC–Gas Chromatograph GC-2010 Plus, Injector–AOC-20i, Autosampler–AOC-20s). Data acquisition and processing was used (GC-MS Real-Time Analysis).

Preparation of Standard and Sample Solutions. The *C. fragile* ethanol extract was dissolved in methanol (HPLC grade) using an ultrasonicator (JAC Ultrasonic JAC-3010) at 6 mg/ml concentration. After extraction, the solution was filtered with 0.2 μm membrane and the fraction of 1 μL filtrate was analyzed using GC-MS system. A standard curve of the phytol solution was prepared at 1 mg/ml (1,000 ppm) with methanol.

GC-MS analysis condition. GC-MS analysis was performed to identify of contents of phytol in *C. fragile*. GC-MS analysis was conducted using DB-5 column (30 mm × 0.25 mm × 0.25 μm). The carrier gas used helium gas (99.999%) eluted with a flow rate of 0.78 ml/min and the split ratio was 1:20. The injector temperature was set at 250°C, column oven programmed from 110°C (isothermal for 2 min), with an increasing rate of 10°C/min to 200°C, then 5°C/min to 280°C, finishing with a 9 min isothermal at 280°C. Ion source electron voltage with 70 eV and Ion source temperature was set was 280°C. Calibration curves, established by standard solution diluted with solvent at each concentration and the limits of detection (LOD) and quantification (LOQ) under the chromatographic conditions, were determined by injecting a series of standard solutions. Chromatograms of each sample were collected under the same condition.

Mice. Wild-type (WT, C57BL/6 strain, 6–8 weeks old, 18–22 g, BW) male mice were obtained from DooYeol Biotech (Seoul, Korea) and acclimated for 1 week. The WT mice were then divided randomly into four groups of five to ten animals each: 1) vehicle group (orally administrated 0.5% low-viscosity CMC), 2) low dose of *C. fragile* treated group (50 mg/kg, BW), 3) high dose of *C. fragile* treated group (100 mg/kg, BW), and 4) ASA treated group (100 mg/kg, BW). The mice were housed in a conventional animal facility with free access to food and water in a controlled temperature and humidity environment under a 12-h/12-h light/dark schedule. The animals were cared for in accordance with the dictates of the National Animal Welfare Law of Korea. The animal experiments (reference number #D-20–057) were approved by the Animal Care and Use Committee of the Korea Institute of Oriental Medicine (KIOM, Daegu, Korea) and performed in accordance with their guidelines.

Isolation of mouse blood platelets. Mouse blood was drawn from isoflurane-anesthetized WT (6–8 weeks old) mice from the inferior vena cava using a one-ninth volume of citrate-dextrose solution (ACD, Sigma). Mouse whole blood was centrifuged at 300 g for 20 min at room temperature (RT) to obtain platelet-rich plasma (PRP). The mouse and human PRP were collected and re-centrifuged at 700 g for 4 min in the presence of 0.5 µM PGE1. The platelet pellet was suspended in HEPES-Tyrode’s buffer containing 10% ACD and centrifuged at 700 g for 5 min. The pellet was re-suspended in HEPES-Tyrode’s buffer, and the final suspensions were adjusted to 3 × 10^8^ platelets/ml.

Platelet aggregation and ATP secretion. The platelet aggregation assay was performed as previously described (Kim et al., 2018). Washed platelets were pre-incubated with 0.01% DMSO or various concentrations of *C. fragile* (30, 50, and 100 μg/ml) or ASA (30, 50, and 100 µM) for 10 min at 37°C and then stimulated with numerous agonists. Platelet aggregation was measured in a 4-channel platelet lumi-aggregometer (Chronolog Corp, Havertown, and PA) at 37°C with stirring at 1,000 rpm. Platelet secretion was monitored as ADP/ATP release by addition of luciferin/luciferase reagent (Chrono-log) to the platelet suspension. In some experiments, mouse platelets were incubated with *C. fragile* (100 μg/ml) prior to treatment with 2 mM EGTA for 10 min at 37°C. In some experiments, mouse platelets were incubated with 100 μg/ml of *C. fragile* aqueous extract for 10 min at 37°C and then stimulated with 0.025 U/ml thrombin or 2 μg/ml Collagen.

TxB_2_ generation assay. Washed platelets were pre-incubated with 0.01% DMSO or various concentrations of *C. fragile* (30, 50, and 100 μg/ml) or ASA (100 µM) for 10 min at 37°C and then stimulated with thrombin (0.025 U/ml) or CRP (0.2 μg/ml) in an aggregometer at 37°C with stirring (1,000 rpm). The reaction was stopped after 5 min by the addition of 2 mM EGTA containing 0.1 M KCl and 5 mM indomethacin for 10 min on ice. The mixture was then centrifuged at 6,000 g for 3 min, and the supernatant was stored at −80°C until analysis. Thromboxane B_2_ (TxB_2_) levels were measured using an enzyme-linked immunosorbent assay kit (Enzo Life Sciences, Farmingdale, NY) according to the manufacturer’s protocol.

Flow cytometric analysis. Washed platelets were pre-incubated with 0.01% DMSO or various concentrations of *C. fragile* (30, 50, and 100 μg/ml) for 10 min at 37°C. Platelets were treated with thrombin (0.025 U/ml) or CRP (0.2 μg/ml) for 5 min at 37°C, followed by incubation with PE-conjugated antibodies against P-selectin or activated αIIbβ3 integrin (JON/A) for 15 min. Cells were analyzed by flow cytometry (Gallios, Beckman Coulter).

Ca^2+^ mobilization. Ca^2+^ mobilization was measured as previously described (Kim et al., 2018). Platelets (1 × 10^8^/ml) were suspended in HEPES-Tyrode’s buffer, pH 7.4 without CaCl_2_ and treated with 0.01% DMSO or *C. fragile* (30, 50, and 100 μg/ml) or ASA (100 µM) for 10 min at 37°C. Cells were incubated with a Ca^2+^ dye (FLIPR Calcium five Assay kit) for 30 min at 37°C in the dark, followed by stimulation with thrombin (0.025 U/ml) or CRP (0.2 μg/ml). Cytosolic Ca^2+^ levels were measured using a spectrofluorometer (Spectramax I3, Molecular Devices) with an excitation wavelength of 485 nm and an emission wavelength of 525 nm Ca^2+^ mobilization was quantified by area under the curve (AUC) and expressed in relative fluorescence units.

Platelet spreading assay. Glass coverslips were coated with human fibrinogen (FG), 100 μg/ml, for 1 h at 37°C and then post-coated with 1% fatty acid-free BSA for 1 h at RT. Mouse platelets, 400 µL (2 × 10^7^/ml), were incubated on the coverslip for 2 h at 37°C in the presence of 0.025 U/ml thrombin. After washing, adherent and spread platelets were fixed with 3% paraformaldehyde, permeabilized with 0.1% Triton X-100, blocked with 0.1% BSA, and stained with 0.1 μg/ml rhodamine-conjugated phalloidin (Sigma). Images were obtained using an Olympus microscope (IX73, Seocho, Seoul, Korea) equipped with 100 x/1.3 NA oil objective lens and recorded with a camera (Andor Zyla sCMOS). Care was taken to image a given fluorochrome at the same settings for all experimental permutations using MetaVue (version 7.8.3.0). Adherent and spreading platelets were monitored in an area of 0.006 mm^2^ and counted in 10 separate fields. The surface area of spread platelets was measured as pixels using ImageJ (v1.52a).

Immunoblotting. Washed platelets were stimulated with thrombin (0.025 U/ml) or CRP (0.2 μg/ml) in the presence or absence of three different concentrations of *C. fragile* (30, 50, and 100 μg/ml) or ASA (100 µM) under stirring conditions (1,000 rpm) in an aggregometer. To measure kinase phosphorylation, platelets (6 × 10^8^/ml) were lysed in an equal volume of 2 x ice-cold lysis buffer (TBS, pH 7.4, containing 2% Triton X-100, 0.1% SDS, 2 mM EDTA, 2 mM Na_3_VO_4_, phosphatase inhibitor cocktail, protease inhibitor cocktail, and 2 mM phenylmethylsulfonyl fluoride) and sonicated. An equal amount of protein (30 µg) was electrophoresed under reduced conditions and immunoblotted, followed by re-probing with different antibodies. In some experiment, spreading platelets were lysed in an equal volume of 2 x ice-cold lysis buffer (TBS, pH 7.4, containing 2% Triton X-100, 0.1% SDS, 2 mM EDTA, 2 mM Na_3_VO_4_, phosphatase inhibitor cocktail, protease inhibitor cocktail, and 2 mM phenylmethylsulfonyl fluoride) into reactions. The band density was measured by densitometry using ImageJ (v1.52a). The level of phosphorylation of each kinase was calculated via normalization of the density of antibodies against the phosphorylated kinases to that of the antibodies against total kinases.

FeCl_3_-induced *in vivo* thrombosis. Mice (10 mice of each group) were orally administered 0.5% low-viscosity CMC and/or *C. fragile* (50 or 100 mg/kg, BW) or ASA (100 mg/kg, BW) twice a day for 3 days, with the administration occurring 30 min before the experiments on the third day. The left carotid artery was isolated, and then filter paper (2 mm diameter) soaked with 10% (460 mM) FeCl_3_ was placed on top of the artery for 2 min. Blood flow was then monitored until 10 min after blood occlusion using a blood flowmeter (AD instruments, Blood flowmeter).

Tail bleeding time. Mice (10 mice of each group) were orally administered 0.5% low-viscosity CMC and/or *C. fragile* (50 or 100 mg/kg, BW) or ASA (100 mg/kg, BW) twice a day for 3 days, with the administration occurring 30 min before the experiments on the third day. Body temperature was maintained at 37°C using a heating pad. Using a sharp razor blade, 5 mm of the tail was removed and held in a 15 ml tube containing 13 ml of PBS prewarmed to 37°C. Tail bleeding was monitored and the time to cessation of blood flow was measured, and after 10 min the bleeding was stopped by cauterization. Blood loss was quantified by measuring the hemoglobin content of blood collected into 13 ml of PBS. After centrifugation, the pellet of red blood cells was lysed with 5 ml lysis buffer (8.3 g/L NH_4_Cl, 1.0 g/L KHCO_3_, and 0.037 g/L EDTA), and the absorbance of the sample was measured at 575 nm.

Statistical analysis. Data analysis was performed using GraphPad Prism 5. Statistical significance was assessed by ANOVA and Dunnett’s test or Tukey’s test for comparisons of multiple groups or Student’s *t-*test for comparisons of two groups. A *p* value of less than 0.05 was considered significant.

## Results

### Extract of *C. fragile* Inhibits Agonist-Induced Platelet Aggregation, ATP Secretion, and TxB_2_ Generation

**FIGURE 1:**
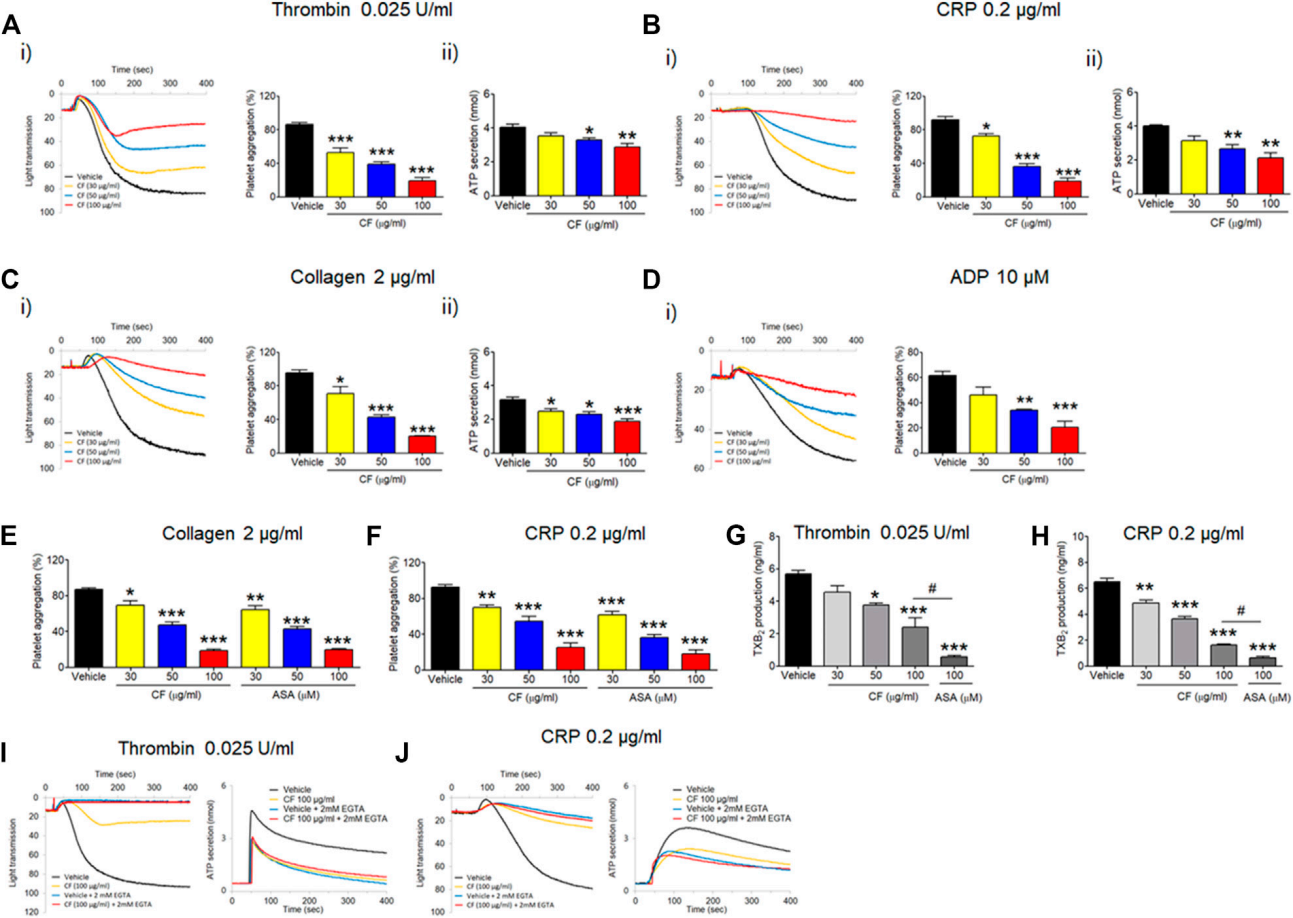
Inhibitory effect of *C. fragile* on platelet aggregation, ATP secretion, and TxB2 production following stimulation with various agonists. Washed mouse platelets were preincubated with various concentrations of *C. fragile* (30, 50, and 100 μg/ml) for 10 min at 37°C and then stimulated with 0.025 U/ml Thrombin **(A)**, 0.2 μg/ml CRP **(B)**, 2 μg/ml Collagen **(C)**, and 10 µM ADP **(D)**. In the ADP-induced aggregation assay, 30 μg/ml of human FG was added to the platelet suspension before ADP stimulation. In some experiments, washed platelets were pretreated with *C. fragile* (30, 50, and 100 μg/ml) or ASA (30, 50, and 100 µM) and then stimulated with 2 μg/ml Collagen **(E)** and 0.2 μg/ml CRP **(F)**. Aggregation was measured for 5 min at 37°C under constant stirring (1,000 rpm) conditions in a platelet aggregometer (Chrono-Log). (i) Platelet aggregation and quantitative graphs. The effect of *C. Fragile* on T_X_B2 generation was measured using a TXB2 ELISA assay kit **(G,H)**. In the ADP secretion, washed platelets were preincubated with various concentrations of *C. fragile* (30, 50, and 100 μg/ml) for 10 min at 37°C before adding a luciferin/luciferase reagent. After the luciferin/luciferase reagent added, platelets were stimulated with 0.025 U/ml Thrombin **(A)**, 0.2 μg/ml CRP **(B)**, and 2 μg/ml Collagen **(C)**. (ii) ATP secretion was measured using a luminometer. In other experiments, mouse platelets were preincubated with a high concentration of *C. fragile* (100 μg/ml) and activated with 0.025 U/ml Thrombin **(I)** and 0.2 μg/ml CRP **(J)** in the presence of 20 mM EGTA. Aggregation and ATP secretion were monitored with a platelet aggregometer. All data represent the mean ± SD (*n* = 5). *:*p* < 0.05, **:*p* < 0.01, and ***:*p* < 0.001 vs. vehicle control after ANOVA and Dunnett’s test.

To investigate the effect of *C. fragile* on platelet function, we first examined platelet aggregation induced by various agonists, including thrombin, CRP, collagen, and ADP. We found that, compared to the vehicle control, platelets treated with *C. fragile* had significantly decreased platelet aggregation induced by the intermediated concentration of thrombin (≤0.025 U/ml), CRP (≤0.2 μg/ml), collagen (≤2 μg/ml), and ADP (≤10 µM), in a concentration-dependent manner (30, 50, and 100 μg/ml) (Figures 1A–D, i). The reduced aggregation of *C. fragile*-treated platelets was similar to the inhibition of platelet aggregation by acetylsalicylic acid (ASA; 30, 50, and 100 µM) in response to collagen (Figure 1E) and CRP (Figure 1F) stimulation. Further, aqueous extraction of *C. fragile* was also investigated to elucidate the inhibitory effect on platelet aggregation. We found that *C. fragile* aqueous extract did not show an inhibitory effect on platelet aggregation (Supplementary Figure S1B). To further confirm the effect of *C. fragile* on platelet function, we examined adenosine triphosphate (ATP) secretion. We observed that compared to the vehicle control, *C. fragile* treatment dose-dependently (30, 50, and 100 μg/ml) inhibited ATP secretion induced by thrombin (0.025 U/ml), CRP (0.2 μg/ml), and collagen (2 μg/ml) (Figures 1A–C, ii). We assessed TxB_2_ generation after stimulation by thrombin (0.025 U/ml) and CRP (0.2 μg/ml). The levels of TxB_2_ were dramatically elevated by both agonists, but treatment with *C. fragile* showed potent inhibition of agonist-induced TxB_2_ generation (Figures 1G,H). These results suggest that *C. fragile* plays a crucial role in stimulating platelet aggregation, ATP secretion, and TxB_2_ generation.

To further examine the way in which *C. fragile* regulates ATP secretion, *C. fragile*-pretreated mouse platelets were incubated with 2 mM ethylene glycol-bis(β-aminoethyl ether)-*N*,*N*,*N*′,*N*′-tetraacetic acid (EGTA) to prevent the interaction of FG with activated αIIbβ3 integrin and then stimulated with thrombin (0.025 U/ml) and CRP (0.2 μg/ml). Neither the vehicle nor the *C. fragile-treated* platelets aggregated but released an equal amount of ATP (Figures 1I,J). Since ATP secretion is only derived from agonist-induced platelet activation in the presence of EGTA (Kim et al., 2013), these results suggest that *C. fragile* reduces the interaction of FG with activated αIIbβ3 integrin.

### *C. fragile* Inhibits P-Selectin Exposure, αIIbβ3 Integrin Activation, and Ca^2+^ Mobilization During Cell Activation

**FIGURE 2:**
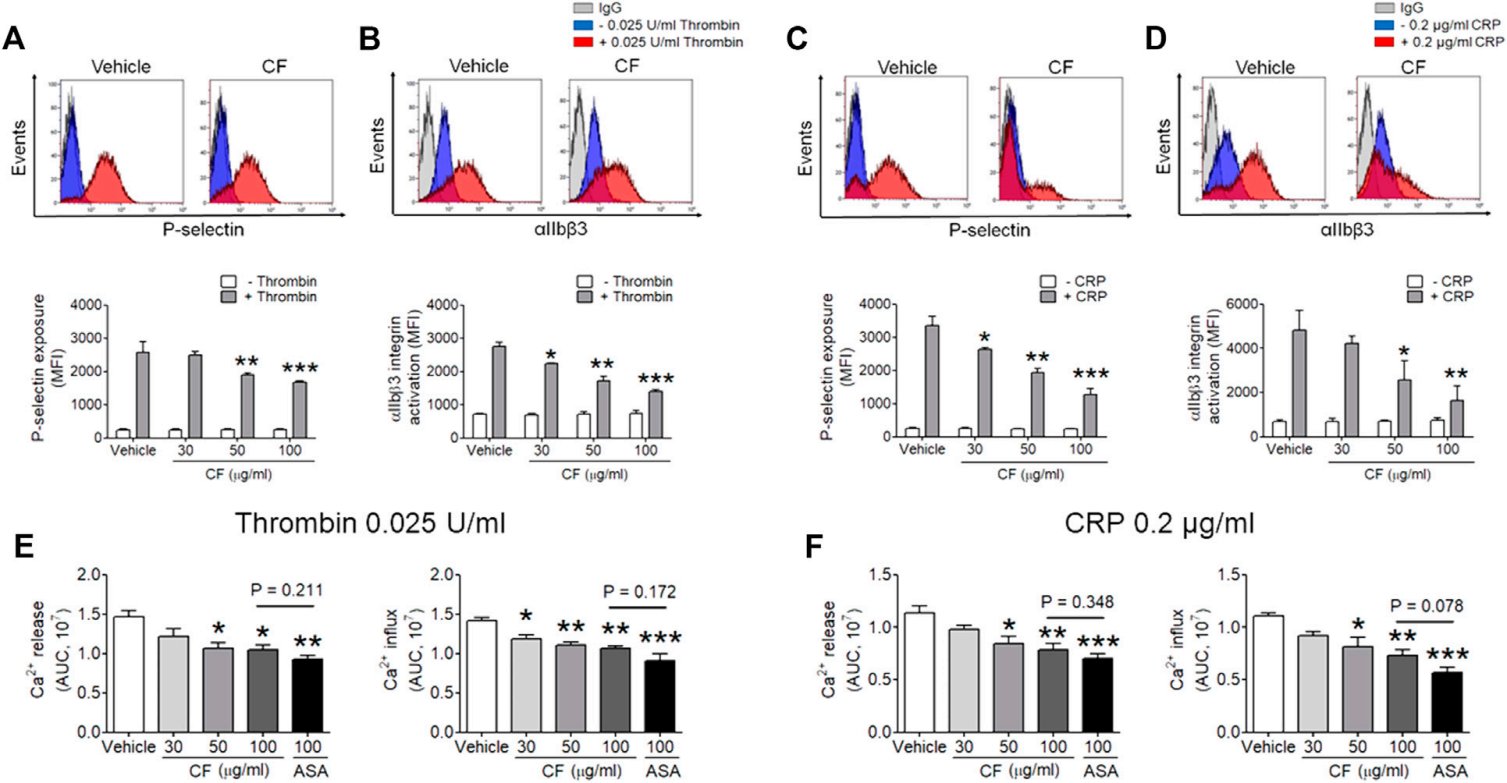
Inhibitory effect of *C. fragile* on P-selectin exposure, αIIbβ3 integrin activation, and Ca^2+^ mobilization during platelet activation. Mouse platelets were pre-treated with the concentration of *C. fragile* (30, 50, and 100 μg/ml), and stimulated with 0.025 U/ml thrombin **(A,B)** or 0.2 μg/ml CRP **(C,D)**. P-selectin exposure and αIIbβ3 integrin activation were analyzed by flow cytometry as described in Methods. The binding of anti-activated αIIbβ3 (JON/A) and anti-P-selectin antibodies to platelets was calculated by the ratio of the geometric mean fluorescence intensity (MFI) value of antibodies to that of control IgG. Data represent mean ± SD (*n* = 5). *:*p* < 0.05, **:*p* < 0.01, and ***:*p* < 0.001 vs vehicle control after ANOVA and Dunnett’s test. In Ca^2+^ mobilization assay, mouse platelets were resuspended in HEPES-Tyrode buffer without 1 mM CaCl_2_ and preincubated with various concentration of *C. fragile* (30, 50, and 100 μg/ml) or ASA (100 µM) for 10 min at 37°C, and then incubated with a calcium-sensitive dye for 30 min at 37°C in the dark. After treatment with a Ca^2+^ dye, platelets were stimulated with platelets were stimulated with 0.025 U/ml Thrombin **(E)** and 0.2 μg/ml CRP **(F)** for 20 min and 2 mM CaCl_2_ was then added for 20 min and 2 mM CaCl_2_ was then added. Intracellular Ca^2+^ release and influx were measured and quantified by the AUC (arbitrary units). Data represent the mean ± SD (*n* = 5). *:*p* < 0.05, **:*p* < 0.01, and ***:*p* < 0.001 vs. vehicle control and a *p* value between two groups after ANOVA and Turkey’s test.

Next, we sought to investigate whether *C. fragile* plays an important role in platelet activation. We assessed the contribution of *C. fragile* to P-selectin exposure, αIIbβ3 integrin activation, and Ca^2+^ mobilization during cell activation. Treatment with *C. fragile* (30, 50, and 100 μg/ml) significantly inhibited P-selectin exposure (Figures 2A,C), αIIbβ3 integrin activation (Figures 2B,D), and Ca^2+^ mobilization (Figures 2E,F) in response to thrombin (0.025 U/ml) and CRP (0.2 μg/ml) stimulation, in a dose-dependent manner. These results suggest that *C. fragile* plays an important role in regulating platelet activation through its effects on granule secretion, Ca^2+^ mobilization, and αIIbβ3 integrin activation.

### Effects of *C. fragile* on Platelet Spreading and Clot Retraction Assay

**FIGURE 3:**
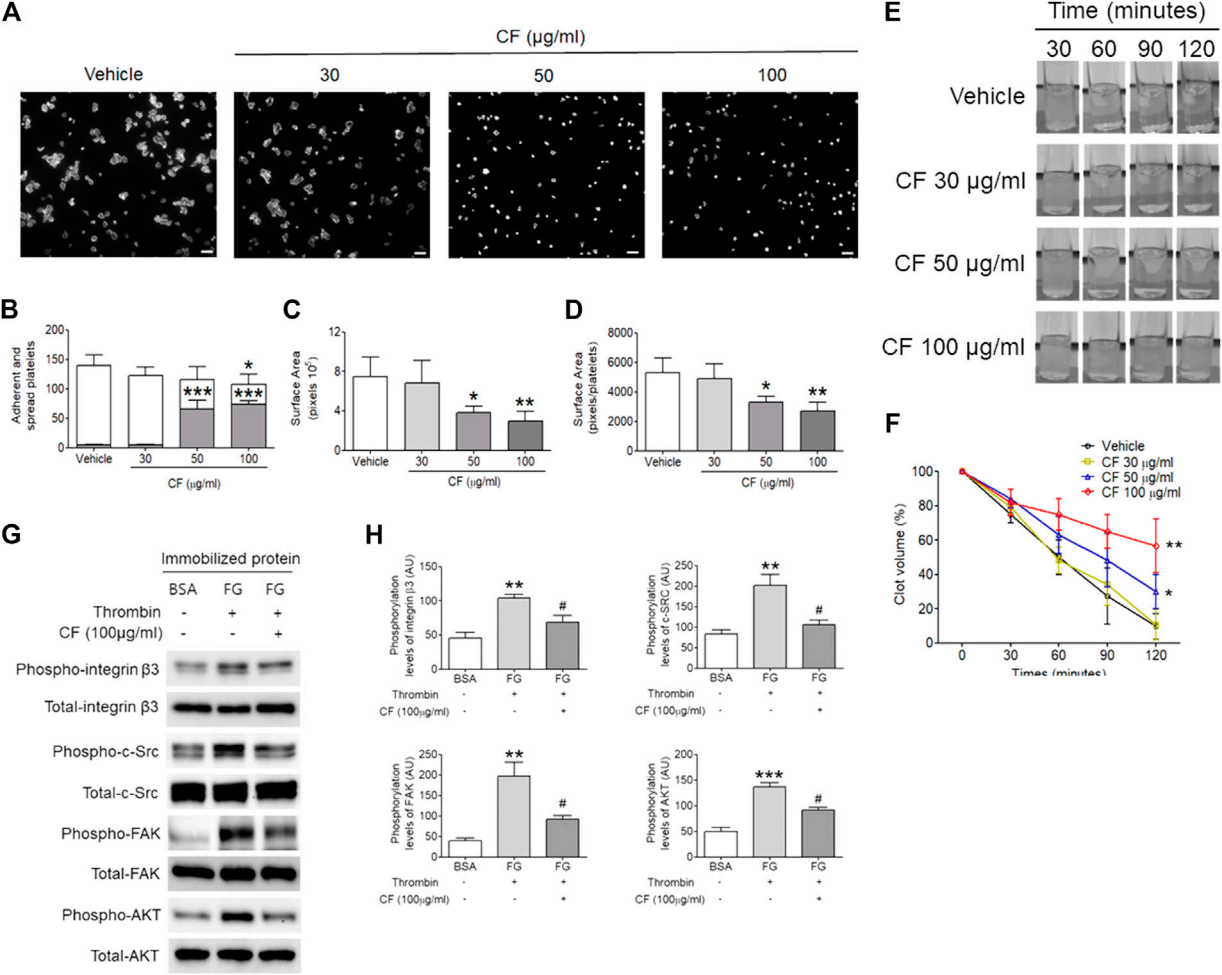
*C. fragile* shows a defective αIIbβ3 integrin-dependent spreading on fibrinogen and fibrin clot retraction. Washed platelets pre-treated with various concentrations of *C. fragile* (30, 50, and 100 μg/ml) or vehicle control (0.01% DMSO) and incubated on FG-coated surfaces for 2 h at 37°C. Adherent and spread platelets were stained with rhodamine-conjugated phalloidin. **(A)** Representative images. Scale bars: 10 µm (white). **(B)** Number of adherents (but not spread, gray bars) and fully spread (white bars). Platelet spreading was analyzed by the surface area **(C)** which was measured by the number of pixels divided by the number of platelets **(D)** in the field. **(E,F)** Mouse platelets were incubated with various concentrations of *C. fragile* (30, 50, and 100 μg/ml) or vehicle control (0.01% DMSO) and fibrin clot retraction was assessed up to 2 h after addition of thrombin, fibrinogen, and CaCl_2_. **(E)** Representative photographs and **(F)** summary data were presented. **(G,H)** The phosphorylation levels of integrin β3, c-Src, FAK, and AKT in lysed spreading platelet. *:*p* < 0.05, **:*p* < 0.01, and ***:*p* < 0.001 vs. vehicle control and #:*p* < 0.05 after ANOVA and Dunnett’s test. Data represent mean ± SD (*n* = 5).

Since *C. fragile* treatment regulated platelet aggregation and ATP secretion through the regulation of the activation of αIIbβ3 integrin, we further examined whether outside-in signaling events were affected by the treatment of *C. fragile* using platelet spreading assays. We found that compared to the vehicle control, *C. fragile* treatment dose-dependently (30, 50, and 100 μg/ml) prevented platelet spreading on immobilized FG (Figure 3A). Although the number of adherent platelets showed a moderate reduction following treatment with a low dose of *C. fragile*, it showed a significant reduction in high-dose *C. fragile* treatment (Figures 3A–D). The lamellipodial actin assembly and surface coverage were markedly reduced by *C. fragile* treatment in a concentration-dependent manner (Figures 3B–D). Platelet spreading and clot retraction assays reflect the processing of early and late-αIIbβ3 integrin outside-in signaling, respectively (Flevaris et al., 2007). The assessment derived from the earliest event of integrin αIIbβ3 mediated outside-in signaling displayed that the activated integrin such as integrin β3 phosphorylation was significantly suppressed by *C. fragile* treatment. Besides the inhibitory effect on FAK phosphorylation, *C. fragile* treatment also inhibited the outside-in signaling by negatively regulating the Src-AKT signaling pathways (Figures 3G,H). Further, we investigated the effect of *C. fragile* on clot retraction. Consistent with the ablated platelet spreading, *C. fragile* showed a significant impairment of clot retraction compared to vehicle control (Figures 3E,F). These results suggest that the effects of *C. fragile* are likely limited to early and late-αIIbβ3 integrin outside-in signaling and that these effects may occur through the regulation of the ligand-binding activity of αIIbβ3 integrin.

### *C. fragile* Plays an Important Role in Regulating αIIbβ3 Integrin Outside-in Signaling

**FIGURE 4:**
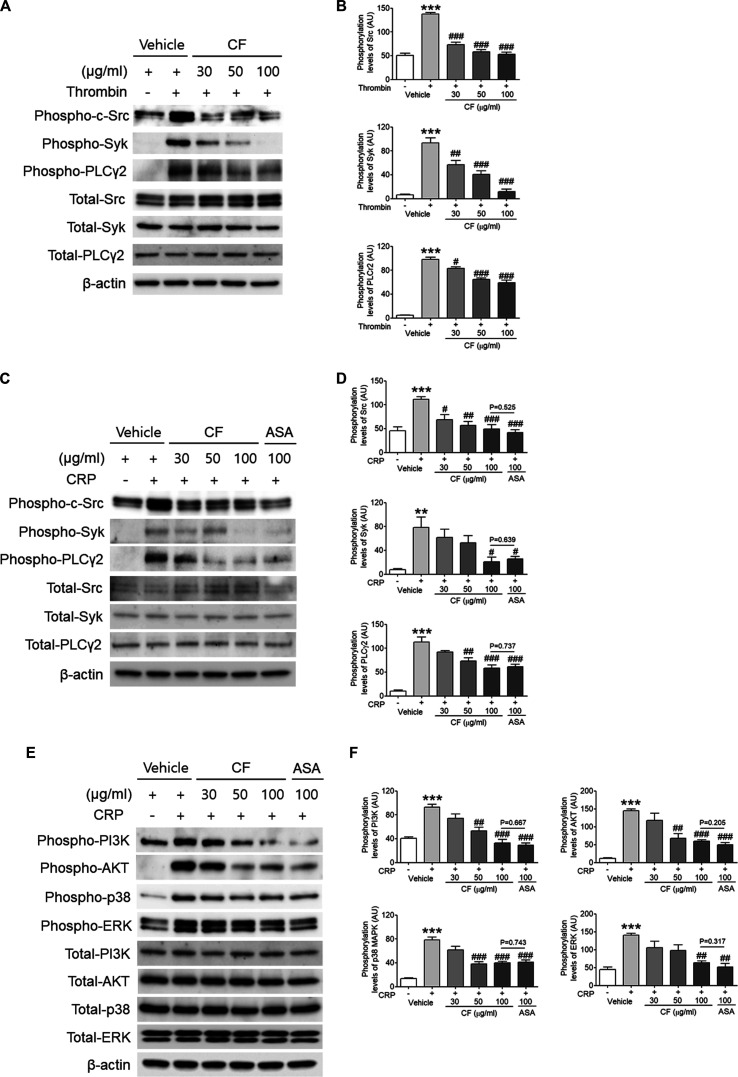
*C. fragile* attenuated phosphorylation of c-Src, Syk, PLCγ2, PI3K, AKT, p38, and ERK after thrombin and CRP stimulation. Mouse platelets were pre-treated with various concentrations of *C. fragile* (30, 50, and 100 μg/ml), and stimulated with 0.025 U/ml Thrombin **(A,B)**. In some experiments, washed platelets were pretreated with *C. fragile* (30, 50, and 100 μg/ml) or ASA (100 µM) and then stimulated with 0.2 μg/ml CRP **(C–F)**. Equal amounts (30 µg) of cell lysate protein were immunoblotted to determine specific inhibition of c-Src, Syk, PLCr2, PI3K, AKT, p38, and ERK phosphorylation. Representative blots **(A,C,E)**. Quantitative graphs **(B,D,F)**. Data represent the mean ± SD (*n* = 5). **:*p* < 0.01 and ***:*p* < 0.001 vs. vehicle control (unstimulated) after Student’s *t*-test, and. #:*p* < 0.05, ##:*p* < 0.01, and ###:*p* < 0.001 vs. vehicle control (stimulated) after ANOVA and Turkey’s test.

The activation of αIIbβ3 integrin outside-in signaling leads to the phosphorylation of Src, Syk, and PLCγ2 (Wonerow et al., 2003; Suzuki-Inoue et al., 2007a; Battram et al., 2017). Therefore, we measured the phosphorylation status of these signaling intermediates. Consistent with the defective platelet spreading and clot retraction, *C. fragile*-treated platelets exhibited a significant reduction of Src, Syk, and PLCγ2 phosphorylation following thrombin (Figures 4A,B) and CRP (Figures 4C,D) stimulation compared with that of vehicle control-treated platelets. These results demonstrated that *C. fragile* treatment has a specific role in the regulation of platelet functions involving the c-Src-Syk-PLCγ2 signaling pathway.

Members of the PI3K-AKT-mitogen-activated protein kinases (MAPK) family, p38, and extracellular signal-regulated kinase (ERK) have been reported to play a pivotal role in the integrin outside-in signaling pathway (Watanabe et al., 2003; Flevaris et al., 2009). We, therefore, investigated the underlying molecular mechanism of the inhibitory effect of *C. fragile* on platelet activation. Biochemical analysis using platelet lysates indicated that compared with vehicle control, pretreatment of platelets with *C. fragile* significantly reduced the phosphorylation of PI3K, AKT, p38, and ERK following CRP stimulation (Figures 4E,F). These results suggested that *C. fragile* might function as a negative regulator of PI3K-AKT-MAPK signaling, to inhibit agonist-induced platelet activation. These findings support the hypothesis that *C. fragile* has a pivotal role in the regulation of platelet integrin αIIbβ3 outside-in signaling.

### *C. fragile* Inhibits *in vivo* FeCl_3_-Induced Thrombus Formation, but Not Hemostasis

**FIGURE 5:**
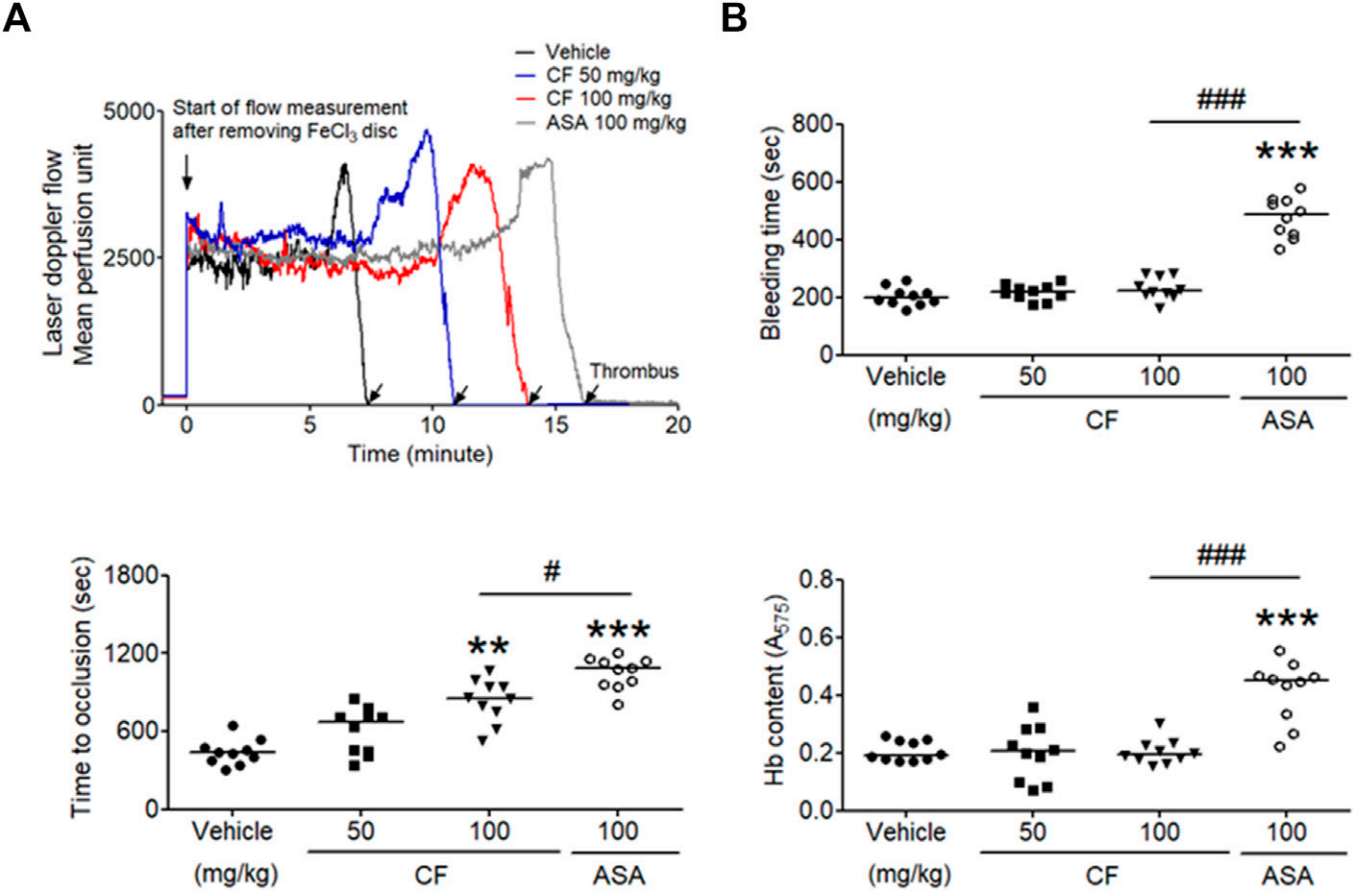
*C. fragile* delayed FeCl_3_^−^ induced arterial thrombus formation but not hemostasis. FeCl_3_-induced arterial thrombus formation was performed as described in Methods. After oral administration of 0.5% low-viscosity CMC and/or *C. fragile* (50 or 100 mg/kg, BW) or ASA (100 mg/kg, BW) twice a day for 3 days **(A)**, the mouse carotid artery was treated with 10% FeCl_3_ for 2 min, and blood flow traces were monitored until stable occlusion took place. Horizontal bars represent the median occlusion time (*n* = 10). In the bleeding time assay, tails of the vehicle (circle), a low dose of *C. fragile* (50 mg/kg, square), a high dose of *C. fragile* (100 mg/kg, reverse triangle), and ASA (open circle) treated mice were amputated **(B)**, and the bleeding time and Hb content were monitored as described in Methods. Horizontal bars represent the median of occlusion and bleeding times for each group of animals (*n* = 10). **:*p* < 0.01 and ***:*p* < 0.001 vs. vehicle control and #:*p* < 0.05 and ###:*p* < 0.001 between two groups after ANOVA and Turkey’s test.

The FeCl_3_-induced vascular injury model has been widely used to study thrombogenesis (Eckly et al., 2011). We investigated whether treatment with *C. fragile* had an inhibitory effect on FeCl_3_-induced thrombus formation. Thrombus formation was evaluated using 10% (460 mM) FeCl_3_. The carotid artery occlusion time in the oral administration of *C. fragile* (50 and 100 mg/kg, body weight (BW)) was significantly prolonged compared to control (Figure 5A). The oral administration of *C. fragile* (100 mg/kg, BW) showed a blood flow prolongation close to that induced by the positive control (ASA, 100 mg/kg, BW). We investigated whether the oral administration of *C. fragile* (50 and 100 mg/kg, BW) influenced hemostatic function. We observed that tail bleeding times were similar between the *C. fragile*- and control-treated groups (Figure 5B). Blood collected from the site of amputation and quantified by hemoglobin content revealed no difference in blood loss between the *C. fragile-* and control-treated groups (Figure 5B). However, the oral administration of 100 mg/kg ASA led to a much longer bleeding time and increased hemoglobin content than that exhibited in the *C. fragile-*treated mice and the vehicle controls (Figure 5B). These results suggest that *C. fragile* inhibits FeCl_3_-induced arterial thrombosis but not hemostasis *in vivo*.

### Content Analysis of Phytol in *C. fragile* Ethanol Extract

**FIGURE 6:**
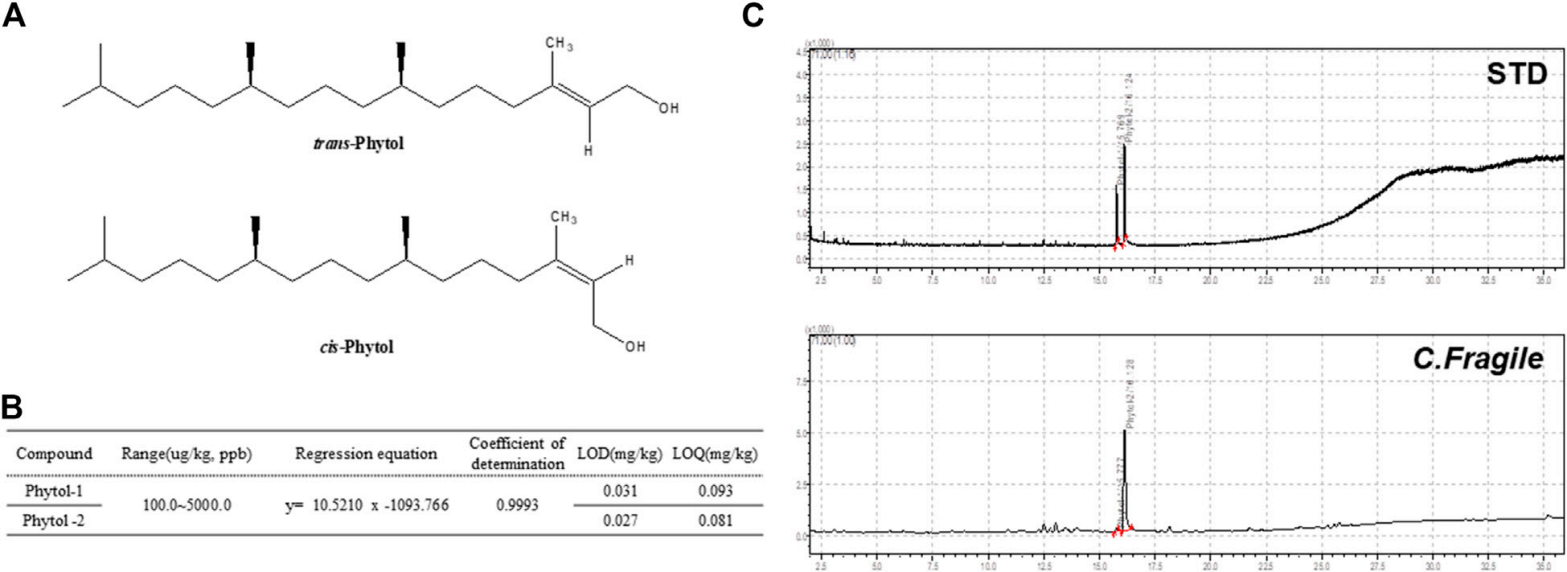
Analysis of *C. fragile* ethanol extract by using GC-MS **(A)** Structure of *trans*-phytol and *cis*-phytol. **(B)** Regression equations, LODs, and LOQs of phytol-1 and phytol-2. **(C)** GC-MS chromatogram of a mixture of phytol isomers standard and *C. fragile* ethanol extract.

The component of the phytol that contributed to the antiplatelet effect in *C. fragile* ethanol extract was determined using GC-MS analysis. Phytol-1 and phytol-2 were successively detected under the GC-MS conditions we described at 15.769 and 16.124 min, respectively, and were found in *C. fragile* ethanol extract at the same retention times, 15.777 and 16.128 min. However, it is not yet clear which peak was *trans*-phytol and which was *cis*-phytol (Figures 6A,C). The calibration curve was y = 10.52101x − 1,093.766, with a coefficient of determination of 0.9993 at injected concentrations of 100–5,000 μg/kg (Figure 6B). The total phytol isomer content was 160.8 mg/kg; it was one of the most abundant compounds in *C. fragile* ethanol extract.

## Discussion

Platelet-derived thrombus formation during vascular damage is the main cause of cardiovascular diseases such as myocardial infarction and ischemic stroke. Although antiplatelet drugs have been widely used in the treatment of thrombotic disorders, these have limited efficacy and concerns have been expressed about their safety (McFadyen et al., 2018). Hence, the development of new antiplatelet drugs is required for the improved treatment of thrombotic disorders. Marine algae could be good therapeutic agents because they are a source of natural derivatives and edible nutrients and a potential source of bioactive compounds, so the risk of adverse effects is reduced. However, few studies have reported on some marine algae and their probable antithrombotic effects (Trento et al., 2001). In the present study, therefore, we evaluated the antithrombotic properties of *Codium fragile* (Suringar) Hariot, which has been used as a therapeutic agent for its anti-inflammatory and antioxidant effects in traditional medicine (Lee et al., 2013; Dilshara et al., 2016). We demonstrated that treatment with *C. fragile* inhibits thrombus formation *in vivo* and *in vitro*. Platelet activation and aggregation following stimulation with various agonists have been markedly reduced by treatment with *C. fragile*, in a dose-dependent manner. Thus, this study indicated an important role for *C. fragile* in platelet activation and aggregation.

Integrins are heterodimeric transmembrane proteins expressed on the cell surface. They act as adhesion receptors that trigger intracellular signaling pathways by binding extracellular ligands (Hynes, 2002). On the surface of platelets, integrin αIIbβ3 acts as a bidirectional receptor for inside-out and outside-in signaling and plays a pivotal role in initiating downstream signaling that triggers intracellular processes such as platelet spreading, adhesion, clot retraction, and aggregation, which leads to platelet thrombus formation and stabilization (Hynes, 2002; Huang et al., 2019). There is increasing evidence of the importance of αIIbβ3 integrin-mediated outside-in signaling in thrombotic disorders. The current understanding of thrombogenesis suggested that the inhibition of outside-in signaling could be a valuable approach to the development of therapeutics with antiplatelet activities, without causing excessive hemorrhage (Shen et al., 2013; Estevez et al., 2015). Our study demonstrated that treatment with *C. fragile* downregulated integrin αIIbβ3 outside-in signaling, thus diminishing platelet spreading on immobilized FG and clot retraction, which may contribute to the effective inhibition of the development of thrombogenesis.

Due to the wide range of biological activities, natural products have long been used as one of the most important strategies for treating and preventing cardiovascular disease (Al Disi et al., 2015; Shaito et al., 2020). However, it is currently difficult to determine to what extent the *in vitro* effects produced can be extrapolated to the *in vivo* situation (Maaliki et al., 2019). In order to investigate, therefore, the effect of oral administration of *C*. *fragile* on thrombus formation, we used a mouse model of FeCl_3_-induced arterial thrombosis, which has been widely used as an experimental arterial thrombosis model. *C. fragile* prevented thrombotic occlusion due to FeCl_3_-induced artery injury. The tail bleeding time was not significantly higher in mice treated with *C. fragile* than in control mice. These results indicate that *C. fragile* can exert antiplatelet and antithrombotic effects without affecting hemostasis.

Although it is unclear how *C. fragile* regulates platelet responses to all agonists, this regulation is probably due to the different key components of *C. fragile*. In this study, we confirmed the content of the major compound in the extract using GC-MS analysis. Phytol was the major component in *C. fragile* extract, with content of 160.8 mg/kg. Phytol is one of the acyclic diterpene alcohols and is a precursor of a synthetic form of vitamin E. Vitamin E is a fat-soluble vitamin and has shown antiplatelet activity when used in conjunction with aspirin (Celestini et al., 2002). Therefore, we suggest that the antiplatelet activity of *C. fragile* ethanol extract was contributed by the high content of phytol in *C. fragile.* Extract of *C. fragile* has been shown to produce anticoagulant properties *via* the fibrinolytic activities of a biofunctional serine protease. Although it is not known which components of *C. fragile* are important for thrombolytic activity *in vitro* and *in vivo*, we speculate that most polyphenolic compounds might play important roles in platelet function. However, the synergy and additive effects of the individual components of *C. fragile* are still unclear. In order to study the pharmacological action of *C. fragile* and its interactions with different targets, a deeper understating of the pharmacokinetics and efficacy of the key components of *C. fragile* is necessary.

In conclusion, our study found that *C. fragile* effectively attenuated platelet activation and thrombus formation by downregulating αIIbβ3 signaling, without affecting hemostasis. Therefore, it may have the potential to be an antithrombotic agent for the treatment of arterial thrombotic disorders and the prevention of thrombotic diseases.

## Data Availability

The raw data supporting the conclusion of this article will be made available by the authors, without undue reservation.

## Abbreviations

ACD, acid-citrate-dextrose solution; ADP, adenosine diphosphate; ATP, adenosine triphosphate; AKT, protein kinase B; ASA, acetylsalicylic acid; BW, body weight; c-Src, sarcoma tyrosine-protein kinase; CMC, carboxymethylcellulose; CRP, collagen-related peptide; DMSO, dimethyl sulfoxide; EGTA, ethylene glycol-bis(β-aminoethyl ether)-N,N,N′,N′-tetraacetic acid; ERK, extracellular signal-regulated kinase; FAK, focal adhesion kinase; FeCl3, ferric chloride; FG, fibrinogen; GC-MS, gas chromatography-mass spectrometry; MAPK, mitogen-activated protein kinase; PI3K, phosphoinositide 3-kinase; PLCγ2, phospholipase Cγ2; PRP, platelet-rich plasma; PPACK, d-Phe-Pro-Arg-chloromethyl ketone; RT, room temperature; SLP-76, SH2 domain-containing leukocyte protein of 76 kDa; Syk, spleen tyrosine kinase; Vav1, Proto-oncogene vav; U46619, thromboxane A2 analogue
