# Supplementary material for: Extract of Seaweed Codium fragile Inhibits Integrin αIIbβ3-Induced Outside-in Signaling and Arterial Thrombosis
Source: Front Pharmacol. 2021 Jul 2;12:685948. doi: 10.3389/fphar.2021.685948 (PMC8283197; doi:10.3389/fphar.2021.685948)
Supplement: Supplementary file 1 [file Presentation1.PPTX]

## Slide 1
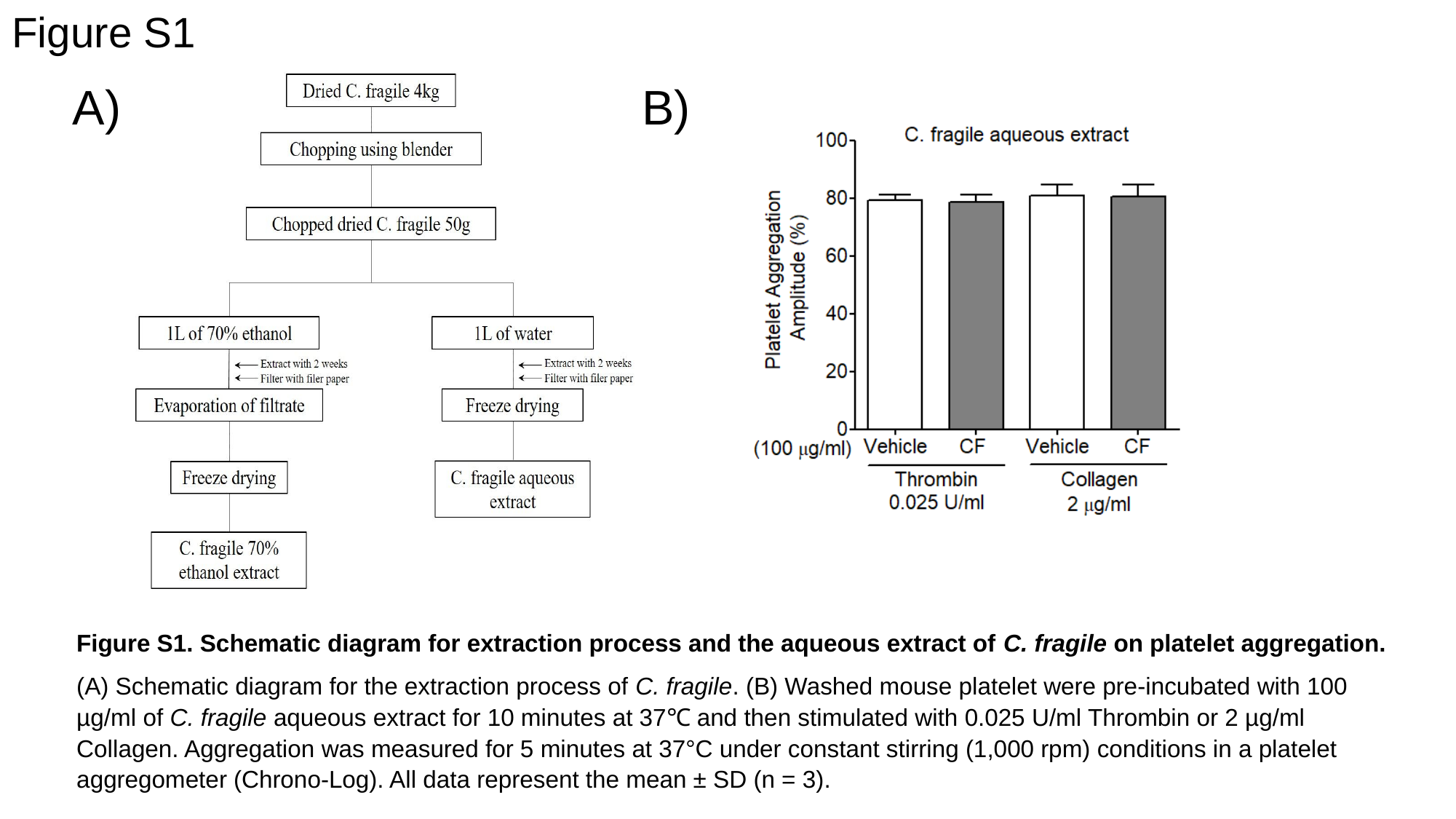

Figure S1
A)
B)
Figure S1. Schematic diagram for extraction process and the aqueous extract of C. fragile on platelet aggregation.
(A) Schematic diagram for the extraction process of C. fragile. (B) Washed mouse platelet were pre-incubated with 100 µg/ml of C. fragile aqueous extract for 10 minutes at 37℃ and then stimulated with 0.025 U/ml Thrombin or 2 µg/ml Collagen. Aggregation was measured for 5 minutes at 37°C under constant stirring (1,000 rpm) conditions in a platelet aggregometer (Chrono-Log). All data represent the mean ± SD (n = 3).

## Slide 2
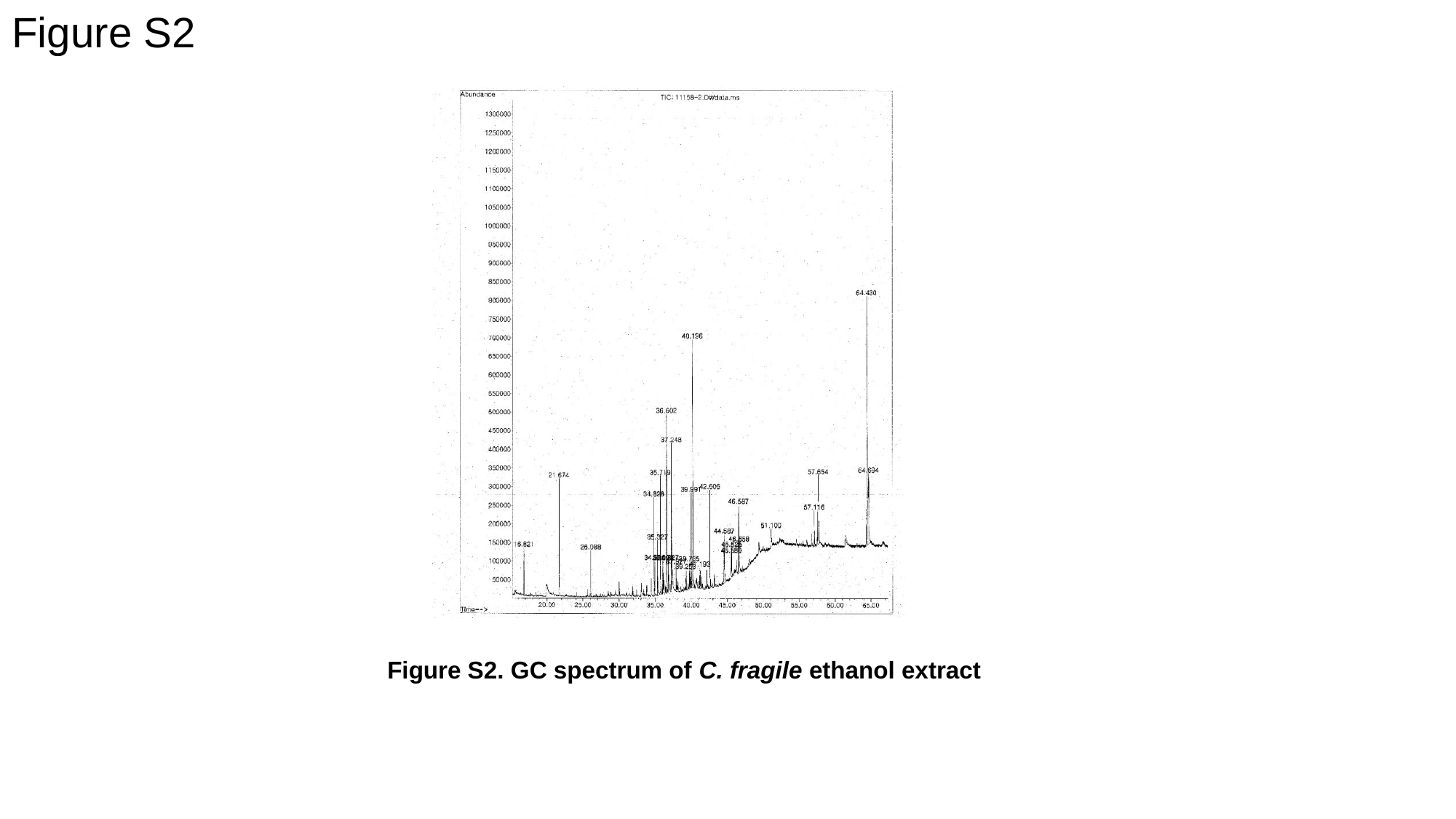

Figure S2
Figure S2. GC spectrum of C. fragile ethanol extract

## Slide 3
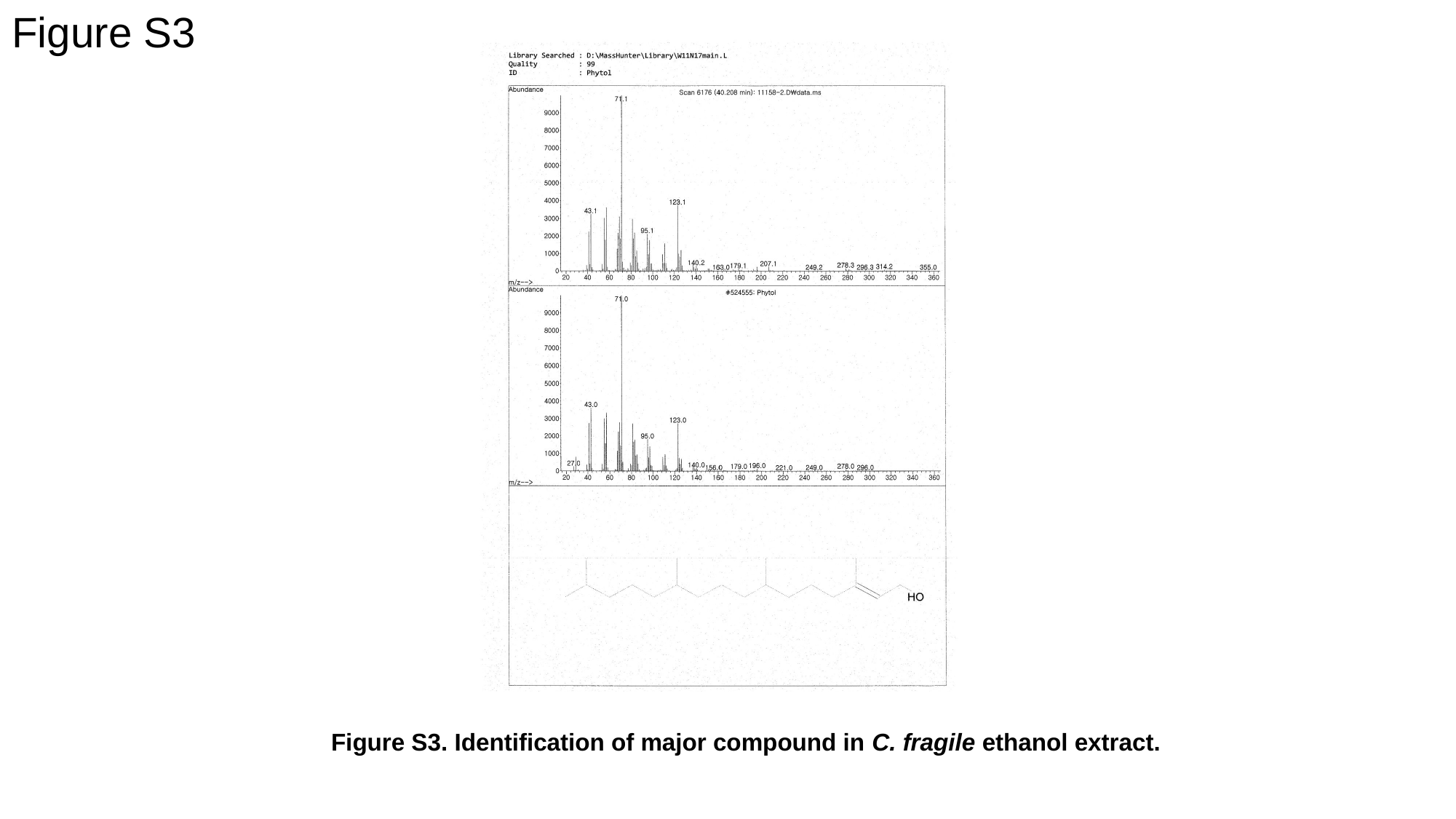

Figure S3
Figure S3. Identification of major compound in C. fragile ethanol extract.
